# 

**Published:** 1916-07-08

**Authors:** 


### Guy's Hospital.

The annual report of Guy's Hospital is for reasons of economy, both in time and money, rather diminished in size, but although some familiar features are omitted, all the information that must be contained in a report, and which the subscribers have a right to receive, is included. The treasurer states that both directly and indirectly the use of Stephen ward as an Officers' Section, with forty-eight cubicles, has contributed towards an unusual variation as regards the statistics concerning in-patients in general. The average stay has lengthened considerably on the medical side, and slightly for surgical cases, therefore the number of in-patients treated was less in 1915 than in the previous year. The number of patients sent to convalescent homes has risen from 928 to 1,287, and a small decrease was observed in practically every department of out-patients. The massage department, reorganised in 1914, when non-resident pupils were admitted, continues its useful work, and last year dealt with 25,000 in- and out-patients, of which 2,000 were treated by Swedish remedial exercises. The total ordinary income (including £5.296 patients' payments) was £71,307, and legacies amounted to £7,339. The total expenditure was £83,448, and there was, consequently, an adverse balance of £4,801. If generally the public knew how this ancient hospital has kept itself abreast of the times as regards its buildings and equipment, and understood the excellent work that the charity is carrying on, there would be no question of a deficit in the accounts.

### That fortunate Leicester Royal Infirmary.

The Leicester Royal Infirmary has received notification from the Treasury Solicitor that, acting under an Order of the Court, the Public Trustee, as the administrator of the estate of the late Mrs. S. L. S. Geldart, has been authorised to pay to the institution a sum of £2,500 from the residuary estate of the testatrix for the general purposes of the institution. The institution also benefits by a legacy of £100 and a fifth share of the residuary estate of the late Miss S. A. Yateman.

### PASSING EVENTS AND TOPICS.

### Sir James Goodhart's Will.

Sir James F. Goodhart, Bart., M.D., F.R.C.P., left unsettled estate of the gross value of £62,217.

### Under Royal Patronage.

Her Majesty the Queen has consented to become Patroness of the Incorporated Society of Trained Masseuses.

### Hospital Sunday.

The amount received to date at the Mansion House for the Metropolitan Hospital Sunday Fund, 1916, collections is £30,000.

### Royal Salop Infirmary.

Mr. William Swire has been elected Treasurer for the coming year, and Mr. G. Butler Lloyd, M.P., has been re-elected Deputy-Treasurer.

### A Hospital's Diamond Jubilee.

The Great Northern Central Hospital celebrated its diamond jubilee on June 28. This hospital has treated over 1,000 soldiers and placed 150 beds at their service.

### Sir Savile Crossley's New Title.

The peerage of Sir Savile Crossley, the well-known hon. secretary of King Edward's Hospital Fund, announced among the Birthday Honours is gazetted as follows: "The Right Hon. Sir Savile Brinton Crossley, Bt., K.C.V.O., Baron Somerleyton of Somerleyton in the County of Suffolk."

### Employment of the Blind.

The Blind Employment Factory, Waterloo Road, S.E., appeals for funds to enable forty skilled artisan men and women to support themselves under suitable conditions and supervision. Contributions should be sent to the Rev. St. Clare Hill, M.A., the secretary.

### Another Scottish Women's Hospital Unit.

Three hospitals on foreign service are already being supported by the Scottish Women's Hospital Organisation—one in France under Dr. Frances Ivens, one in Salonika under Dr. Louise M'Ilroy, and one in Corsica under Dr. Mary Blair. Now it is proposed to provide a fourth complete hospital unit, together with ambulance transport for the wounded, to be attached to the newly equipped Serbian army at Salonika.

### Comforts for Indian Prisoners.

The report of the Lady Hardinge Hospital, at Brockenhurst, for Indian soldiers, states that the hospital cared for 2,629 men, with a mortality of only 1.4 per cent., and its expenditure was £1 17s. 4d. a bed per week; of the patients admitted 55.1 per cent. returned to duty. Although the hospital is now closed, the committee of the Indian Soldiers' Fund points out that the fund's needs are increasing. It has been supplying clothing and comforts to the Indian Forces in East Africa, Mesopotamia, and Egypt, assisting the 700 Indian prisoners in Germany, and is now hoping to alleviate the lot of the 9,000 Indians interned in Turkey.

### THE COLLEGE OF NURSING, LIMITED.

### Official Notices.

The College of Nursing having now started on its way, the detailed work with which the able secretary, Miss Rundle, and her staff will be faced must necessarily be great and increasing. With a view to diminish correspondence and lighten the detailed work as much as possible we propose to devote a page, or as much space as may be necessary, each week to Official Notices, Answers to Correspondents, and any other matter which the Council or its officers may find helpful to them in lightening the work and getting the administration into order. This week we give a number of official facts and information relating to the

### OBJECTS.

The College of Nursing has been founded with the following objects: 1. To organise the nursing profession. 2. To secure State registration for the trained nurse. 3. To make and maintain a Register of trained nurses, and by this means to protect the public. 4. To protect the interests of trained nurses. 5. To raise and maintain the standard of training. 6. To establish a uniform curriculum of training, and one portal examination. 7. To establish lectureships, scholarships, and in every way to promote the advancement of the nursing profession.

### Application for Registration.

Every certificated trained nurse should apply at once for registration by the College of Nursing:

1. Because the Council of the College of Nursing has drafted a "Nurses' Registration Bill," which provides that the Register already formed by the College of Nursing shall be the first Register under the Act. If, therefore, you are on the College Register you will, automatically and without further fee, be placed upon the State Register when the "Nurses' Registration Bill" is passed.

2. Because every nurse who is placed upon the College Register is *ipso facto*, and without further fee, a member of the College, and is entitled to vote for the election of the Council.

3. Because the greater the number of trained nurses on the College Register, the sooner will the Government be inclined to grant facilities for the passing of the Bill.

4. Because the College of Nursing is a democratic body, being governed by trained nurses themselves, as two-thirds of the Council must be elected by members of the College. Thus every member of the College of Nursing, being entitled to vote, will, through her representatives, assist in the government of the College, and therefore the government of her profession.

5. Because the larger the number of members of the College, the greater will be its power to carry out the objects for which it was founded.

6. Because it is proposed to build a College in a central part of London which shall be the headquarters of the nursing profession, and to form centres in different parts of the kingdom.

7. Because if a member is in need of any advice concerning her profession as a trained nurse, she will receive by the authorities of the College such help and advice as they are able to give.

### Conditions of Registration during Period of Grace for Existing Nurses.

Applicants are required:

(*a*) To be at least twenty-one years of age.

(*b*) To be of good character.

(*c*) To hold a certificate or certificates of three years' training in a Nurse-Training School or Schools recognised by the Council for the purpose of admitting practising nurses to the Register of the College; or

(*d*) To hold a certificate of not less than two years' training in a Nurse-Training School recognised by the Council for the purpose of admitting practising nurses to the Register, followed by at least two years' *bona fide* practice as a nurse; or

(*e*) To produce evidence of training to the satisfaction of the Council, having regard to the date at which the training was taken, followed by at least five years' *bona fide* practice as a nurse.

### Fees.

The fee for registration and membership has been fixed at £1 1s. (one pound and one shilling).

### Forms of Application.

Forms of application for registration and membership can be obtained from the Secretary of the College, 6 Vere Street, Cavendish Square, London, W. The envelope should be marked "Registration," and an addressed and stamped envelope enclosed for reply.

---

### FORM OF APPLICATION FOR REGISTRATION.*

To the Council of the College of Nursing, Limited.

I, (Christian and surnames in full) ................................

(State here whether single, married, or widow. If married or widow, give maiden name, and send copy of marriage certificate) ................................

(Full postal permanent address) ................................

(A change of permanent address must be notified to the Secretary.)

hereby request the Council of the College of Nursing to enter my name upon the Register of Trained Nurses maintained by them. I promise, in the event of my being so registered, and in consideration thereof, to conform in all respects to the rules and regulations from time to time adopted by the Council of the College.

I forward herewith a copy of my certificate of training. (Original certificate not to be sent unless asked for.)

(Name of training school or schools) ................................

Date of certificate ................................

(If no certificate is held by the applicant, explain why)........

The following are the places and dates of my nursing work, and appointments:—

| Places | | Dates Month Year | | Month Year |
|---|---|---|---|---|
| .................................... | From | .................. | to | .................. |
| .................................... | ,, | .................. | ,, | .................. |
| .................................... | ,, | .................. | ,, | .................. |
| .................................... | ,, | .................. | ,, | .................. |
| .................................... | ,, | .................. | ,, | .................. |
| .................................... | ,, | .................. | ,, | .................. |

I hereby declare that the above particulars are in every respect complete and true.

Signature of Applicant ................................

Witnessed by ................................

Date ................................

* Registration includes membership of College.

Give names and addresses of three referees—not relatives—stating how long each has known you:—

................................................................

................................................................

................................................................

................................................................

................................................................

---

**REGISTRATION FEE, £1 : 1 : 0 (one pound and one shilling). To be sent only after the applicant has been notified that her application for Registration is in form for acceptance by the Council.**

Rates of Subscription: Twelve months, 6/6; Six months, 4/-; Three months, 2/-, post free to any address in the United Kingdom. Abroad, Twelve months, 8/8; Six months, 5/-; Three months, 2/6, post free. The Hospital "Index" to Volume LIX., October, 1915 to March 1916, is now ready, price 1½d. per copy, post free. Address The Manager, "The Hospital," The Hospital Building, 28/29 Southampton Street, Strand, London, W.C.

